# Studies on *Trans*-Resveratrol/Carboxymethylated (1,3/1,6)-β-d-Glucan Association for Aerosol Pharmaceutical Applications

**DOI:** 10.3390/ijms18050967

**Published:** 2017-05-03

**Authors:** Antonio Francioso, Riccardo Cossi, Sergio Fanelli, Paola Mastromarino, Luciana Mosca

**Affiliations:** 1 Department of Biochemical Sciences, “Sapienza” University of Rome, Piazzale Aldo Moro, 5, 00185 Roma, Italy; fanelli.sergio@yahoo.it (S.F.); luciana.mosca@uniroma1.it (L.M.); 2 QI Technologies, Via Monte D’Oro 2/A, Pomezia, 00071 Rome, Italy; r.cossi@qitech.it; 3 Department of Public Health and Infectious Diseases, “Sapienza” University of Rome, Piazzale Aldo Moro, 5, 00185 Roma, Italy; paola.mastromarino@uniroma1.it

**Keywords:** aerosol, resveratrol, carboxymethylated glucan, MMAD

## Abstract

A resveratrol/carboxymethylated glucan (CM-glucan) combination is known to inhibit rhinovirus replication and expression of inflammatory mediators in nasal epithelia. The aim of this study was to develop an aerosol formulation containing an association of the two molecules which could reach the lower respiratory tract. Mass median aerodynamic diameter (MMAD) of a resveratrol/CM-glucan combination was lower than that shown by resveratrol or CM-glucan alone (2.83 versus 3.28 and 2.96 µm, respectively). The resveratrol/CM-glucan association results in the finest and most monodispersed particles in comparison to the two single components. The association also evidenced lower values for all particle size distribution parameters, suggesting that the pharmacological synergy observed in previous studies may be accompanied by a pharmaceutical one. Moreover, we showed that the CM-glucan matrix did not exert an inhibitory effect on resveratrol nebulization, demonstrating the good suitability of these two molecules in association for simultaneous aerosol volatilization.

## 1. Introduction

Human rhinoviruses (HRV) are the main etiological agents responsible for upper respiratory tract infections in children and adults. They are the leading cause of the common cold and the exacerbation of respiratory diseases such as asthma and chronic obstructive pulmonary disease (COPD). In total, they are responsible for about 50% of viral respiratory infections and half to two-thirds of cases of asthma exacerbation induced by viruses [1].

Noteworthy, experimental and observational studies evidenced that HRV can spread to the lower airway epithelium and act as a lower respiratory tract pathogen [2,3,4,5,6]. Indeed, in-situ hybridization studies demonstrated that HRV replication occurs in vitro in primary Human Bronchial Epithelial Cells (HBECs) and in vivo in bronchial tissue of five out of ten subjects experimentally infected [4]. Similar results were obtained by Gern et al. in eight allergic volunteers experimentally infected with HRV-16 [6]. Rhinovirus RNA was detected by reverse transcription-PCR (RT-PCR) in bronchoalveolar lavage cells from all subjects two to four days after infection. Furthermore, rhinoviruses induced release of proinflammatory mediators (interleukin (IL)-6, -8, and -16 and CCL-5 chemokine) from cultured HBEC (Human Bronchial Epithelial Cells) [4]. Histological analyzes from subjects with infections of the lower respiratory tract by HRV have shown that HRV determines both interstitial and alveolar processes. Indeed, bronchiolitis obliterans with organizing pneumonia [7], interstitial pneumonitis [8,9], acute and chronic inflammation with fibrinopurulent alveolar debris [10] and hyperplasia, and desquamation of alveolar cells [11] have been observed. All these studies confirm that HRV infects the lower airways and induces a proinflammatory response.

**Figure 1:**
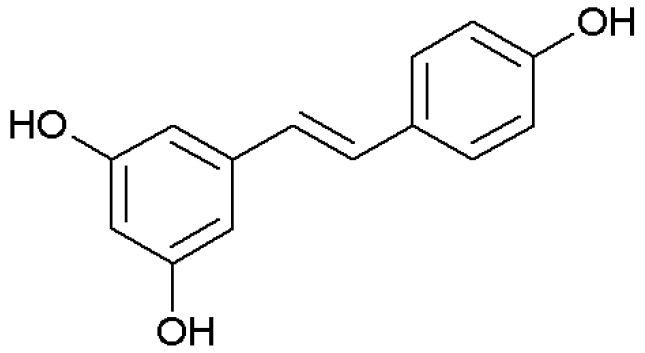
*Trans*-resveratrol.

We recently showed that resveratrol (Figure 1), a polyphenolic phytoalexin produced by several plants, inhibits rhinovirus replication and expression of inflammatory mediators in nasal epithelia, suggesting a potential for application in lower respiratory tract infections, COPD, and asthma [12]. Resveratrol use as a drug has been limited by its poor solubility and scarce bioavailability after oral administration [13]. Therefore, appropriate preparation and delivery remain issues, though an aerosol version would have obvious benefits.

**Figure 2:**
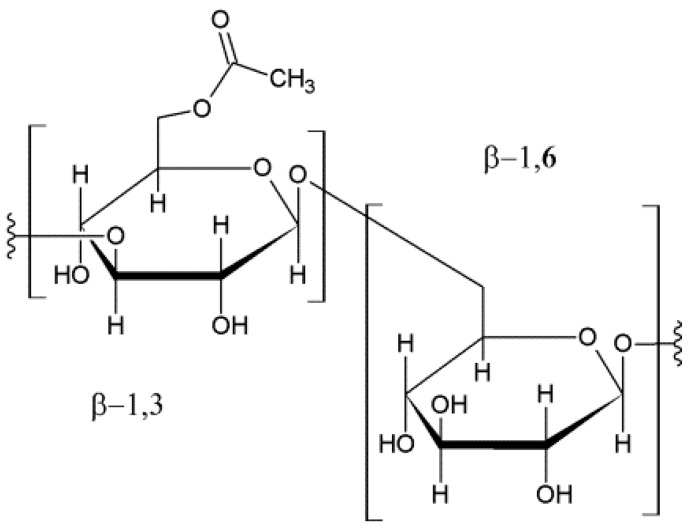
Carboxymethylated-(1,3/1,6)-β-d-glucan (CM-glucan).

It has been recently demonstrated that resveratrol can be stabilized in aqueous solutions when combined with a modified β-glucan; this produces a carboxymethylated-(1,3/1,6)-β-d-glucan (CM-glucan) (Figure 2), which greatly improves the stability of resveratrol in solution without altering its biological activity [12,14].

β-Glucans are high molecular weight polysaccharides largely produced in nature by fungi, cereals, and bacteria. They are endowed with multiple biological functions and are considered as typical “biological response modifiers” as they are able to stimulate the immune system, thus exerting anticancer, antiviral, and wound healing effects [13,14,15,16]. β-Glucans also increase resistance to infections via activation of macrophages/monocytes, neutrophils, and the reticuloendothelial system. They stimulate microbial killing and initiate production of innate immune system components including inflammatory mediators such as tumor necrosis factor (TNF)-α, IL-1, and macrophage inflammatory proteins. β-Glucans are also used in the pharmaceutical industry for their drug delivery properties [17]. The carboxymethylation of these biopolymers confers on them solubility in an aqueous environment without compromising their biological activities, hence, these modified polymers are increasingly being used in pharmaceutical preparations exploiting both their pharmaceutical and their biological properties [14]. Our recent work exploring the antiviral capacity of the combination of resveratrol and CM-glucan has demonstrated that CM-glucan does not affect resveratrol antiviral activity and that the two compounds may act in synergy, exerting anti-inflammatory activity by modulating cytokines production [12]. The synergy between β-glucan and resveratrol was also demonstrated by previous studies performed by Vetvika et al., who demonstrated that this combination stimulates the immune system and that it potentiates the anticancer properties compared to the effect exerted by the single substances [18,19,20].

The effectiveness of our new formulation containing the combination of resveratrol/CM-glucan was tested as a nasal spray in a clinical trial in asthmatic children and was found to reduce the severity and recurrence of upper respiratory tract infections [15,16,21]. Based on this evidence, we developed an aerosol formulation for inhalation to reach the bronchial epithelia for the optimal anti-inflammatory and antiviral targeted activity. On this topic, a recent paper reported on the preparation of resveratrol as a dry powder for inhalation for the treatment of inflammatory lung diseases [22]. In this paper, we present evidence that the resveratrol/CM-glucan combination may be suitable for the administration via inhalation, suggesting that it could be useful in lower respiratory tract infections, COPD, and asthma.

## 2. Results and Discussion

Our previous work demonstrated that the association of resveratrol with CM-glucan is more stable in the long term in comparison to aqueous formulations of resveratrol alone [14]. In this paper, we evaluated the feasibility of an aerosol application of the resveratrol/CM-glucan formulation by studying some aspects of the nebulization process. First, we analyzed the aerosol MMAD (mass median aerodynamic diameter) of resveratrol, CM-glucan, and the combination of both compounds in aqueous liquid formulations. The three samples showed some differences as to the mean diameter, width of the distribution, and shape of the distribution. Data in Table 1 show that the CM-glucan and resveratrol alone had MMAD values higher than that of the complex of the two molecules (2.83 µm for the complex versus 3.2 and 2.96 µm for resveratrol and CM-glucan, respectively). Furthermore, the geometric standard deviation (GSD) is smaller for the complex of the two molecules compared with the single components. The GSD, i.e., the Geometric Standard Deviation, is calculated on at least 60,000 particles and is a dimensionless factor that, multiplied and divided by the geometric mean of the data set, defines an interval containing 68.27% of the whole distribution. This means that the particle distribution of the complex is narrower than that of the single components, as also confirmed by the smaller Span value.

The resveratrol/CM-glucan sample showed the presence of the finest and also the most monodispersed particles in comparison to the two single components that were more heterogeneous and polydispersed, as also evidenced from D90, D10, and D50 values, underling that the association could be more useful for aerosol applications with respect to the single bioactive molecules. Furthermore, only the D90 value for the resveratrol/CM-glucan association is below 5 µm, underlying that more than 90% of the product can reach the bronchial alveoli. These features suggest that the already demonstrated pharmacological synergy could be accompanied by a pharmaceutical one [12,13,14,15,16].

**Figure 3:**
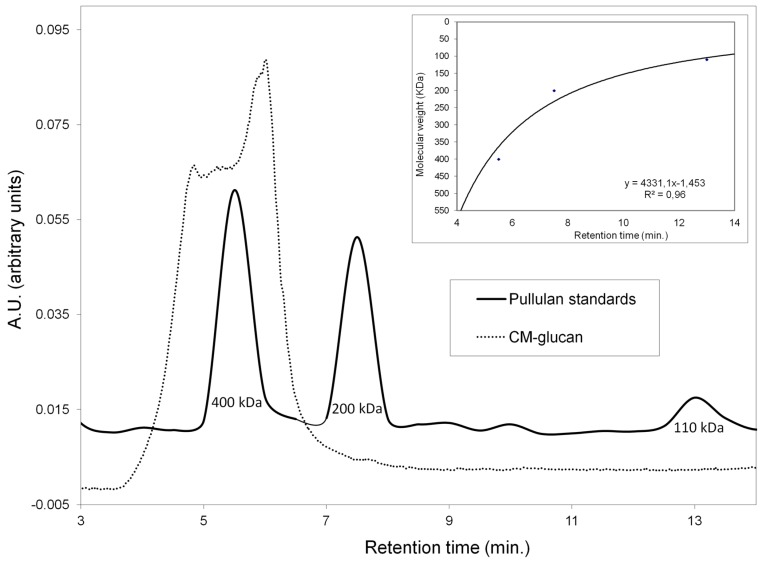
Pullulans (black line) and CM-glucan (dashed line) gel filtration chromatography (GFC) chromatograms.

Figure 3 shows the chromatographic profile of the CM-glucan aqueous solution overlapped to that of the Pullulan molecular weight standards profile (400–200–110 kDa). This analysis revealed that the CM-glucan molecular weight is ~250–550 kDa. Notably, this profile did not change during the aerosol nebulization process, when CM-glucan was nebulized alone, nor in the presence of resveratrol; this also indicates that the different molecular weight species were able to be aerosolized with the same efficiency.

**Figure 4:**
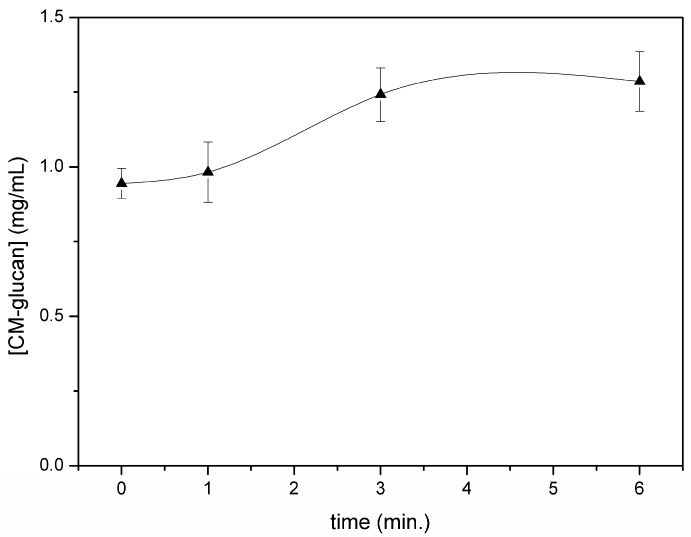
CM-glucan residual amount after various nebulization times of resveratrol/CM-glucan formulation.

A slight increase in the concentration of the initial material in the chamber was observed within 6 min of nebulization (Figure 4). This slight increase could be due to the faster nebulization of the solvent in the initial steps of the aerosolizing process. Regarding the residual content of CM-glucan after complete aerosol nebulization (10 min), data showed that only 3% of the initial material remained in the nebulizing chamber, indicating that the polymer shows a very good propensity to be sprayed in a homogeneous manner from a quantitative point of view.

**Figure 5:**
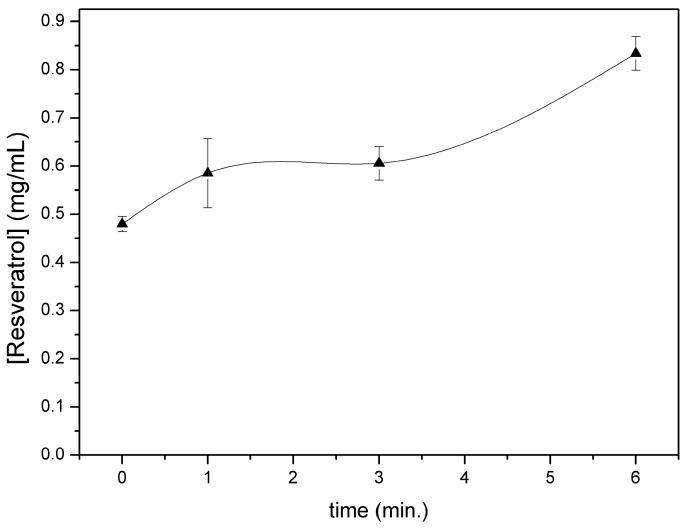
Resveratrol residual amount after different times of nebulization of resveratrol/CM-glucan formulation.

**Figure 6:**
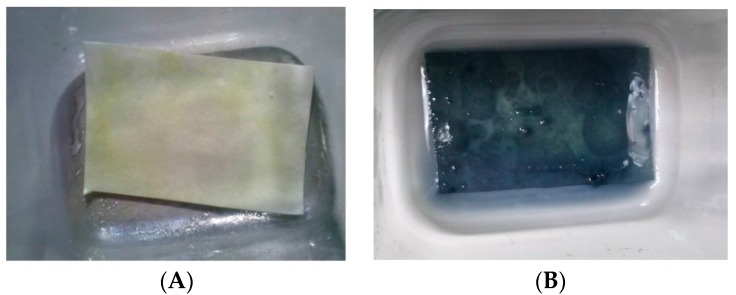
Folin-Ciocalteau staining of tissue paper filters after complete nebulization of CM-glucan formulations in the absence (**A**) or presence (**B**) of resveratrol.

Figure 5 shows resveratrol content in the chamber after different nebulization times. As already observed for CM-glucan, the polyphenol showed a slight increase in its concentration in the initial phases of aerosol nebulization, reaching a maximum after 6 min. However, in addition, the residual content in the aerosol chamber after complete nebulization was low (less than 10% of the initial amount). After complete aerosolization, we also verified resveratrol presence in nebulized material by entrapping it in tissue paper filters and by revealing it with Folin–Ciocalteau reagent, confirming the effective nebulization of the polyphenol (Figure 6). These data indicate that nebulization of resveratrol was efficient and also that the CM-glucan matrix did not exert an inhibitory effect on its nebulization, demonstrating the good suitability of these two molecules in association for simultaneous aerosol volatilization. These features make this association helpful for applications in pharmaceutical aerosols to be used in virus-induced respiratory diseases such as COPD and asthma.

**Figure 7:**
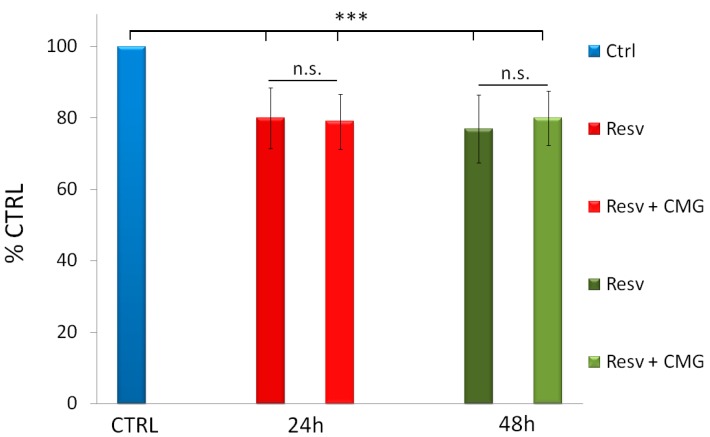
HeLa cell viability after 24 or 48 h incubation with 100 µM resveratrol (Resv) in the presence or in the absence of 0.05 mg/mL CM-glucan (CMG). Values are mean ± S.D. and expressed as percent versus control cells. *** *p* < 0.001 vs. CTRL.

In order to evaluate whether the biological activity of resveratrol was affected by the combination with CM-glucan, we tested the effect of resveratrol on HeLa cells, in the presence or absence of CM-glucan. It is well known that resveratrol concentrations higher than 30 µM may exert an antiproliferative effect on cells. Resveratrol at 100 µM exerted a cytotoxic effect on HeLa cells, decreasing cells viability by about 20% after 24 and 48 h incubation. Notably, the association of resveratrol with CM-glucan did not alter cell proliferation in a statistically significant manner (Figure 7).

## 3. Materials and Methods

### 3.1. Reagents

*Trans*-resveratrol was purchased from Shangai Novanat Co., Ltd. (Shanghai, China) and CM-glucan from Nutraceutica (Bologna, Italy). HPLC grade solvents used for chromatographic analyses were purchased from Carlo Erba Reagents (Milan, Italy). All other reagents were analytical grade products from Sigma-Aldrich (Milan, Italy).

### 3.2. Samples

Formulation of resveratrol/CM-glucan contained 1 mg/mL of CM-glucan and 0.5 mg/mL of resveratrol in saline solution. Final resveratrol/CM-glucan ratio was 1:2 (*w*/*w*) and the pH of the formulation was around 5.5–6.0. CM-glucan (0.1% *w*/*v*) was dissolved in 0.9% NaCl and magnetically stirred for 15 min at room temperature. To an aliquot of this solution, resveratrol was added at a final concentration of 0.05% (*w*/*v*). Aqueous solutions of CM-glucan and CM-glucan containing resveratrol were nebulized by an available commercial aerosol apparatus “MicroLife”. Resveratrol and CM-glucan quantitative analyses were performed by reverse-phase high performance liquid chromatography (RP-HPLC) and gel filtration chromatography (GFC), respectively, by sampling the solutions contained in the nebulizer chamber at 1, 3, and 6 min during aerosolization. After complete nebulization (about 10 min) the dry residue in the chamber was resuspended in 1 mL of mobile phase and analyzed by HPLC.

### 3.3. Particle Size Distribution

Aerosolized samples were fluxed onto the Aerosizer apparatus (Time of Flight Aerosol Beam Spectrometry “Aerosizer LD”, Amherst Process Instruments Inc., Hadley, MA, USA) at 2 L/min. We used a run longer than 40 s from a sampling chamber of 13 L where aerosol has been introduced for about 2 s. Droplets entered into the measuring chamber through a sonic nozzle in which air moves at about mach2 in a choked flow regime, where gas speed is constant in the supersonic region and insensitive to pressure fluctuation (pressure fluctuation can change supersonic region length but not speed inside). As long as the measuring chamber is below 250 mbar, the two laser beams are within the supersonic region, so drag force applied to particles is known and stable. During the time in which particles are in the nozzle, they are submitted to a drag force. Time of flight is obtained at the end of the nozzle by detecting 90 degrees scattering on two parallel HeNe laser beams by two photomultipliers. Time of flight aerodynamic mass and then diameter (MMAD) is obtained from Aerosizer software, based on a calibration curve, factory validated with NIST traceable standards (National Institute of Standards and Technology). A chamber was used to avoid influence from ambient air and to limit evaporation. Limited time in aerosol introduction avoided saturation of the chamber and analyzer overload. Sample stream was generated by a low pressure chamber inside the instrument (at approx. 80 mbar) which sucks air and sample from the sampling chamber [23,24,25]. To obtain statistically relevant data, at least 60,000 particles were analyzed for each sample.

### 3.4. RP-HPLC Resveratrol Analyses

The HPLC consisted of a Waters apparatus equipped with a 600 pump and pump controller, a Waters autosampler mod. 717, and a UV-Visible Photodiode array detector, mod 2996. For resveratrol determination, an isocratic chromatographic method was used. Mobile phase was composed by 80% of solvent A (10% acetic acid) and 20% of solvent B (acetonitrile). The HPLC column was a Symmetry C18 reverse phase (3.9 mm × 150 mm, 5 μm particle size, with a 10 mm guard column of the same material) and flow rate was 1 mL/min. Resveratrol quantitation was performed by peak area integration at 306 nm. Before the analysis, 20 μL of each sample was diluted 1:10 in mobile phase, filtered onto 0.2 µm filters, and then 50 µL were injected onto the column.

**Figure 8:**
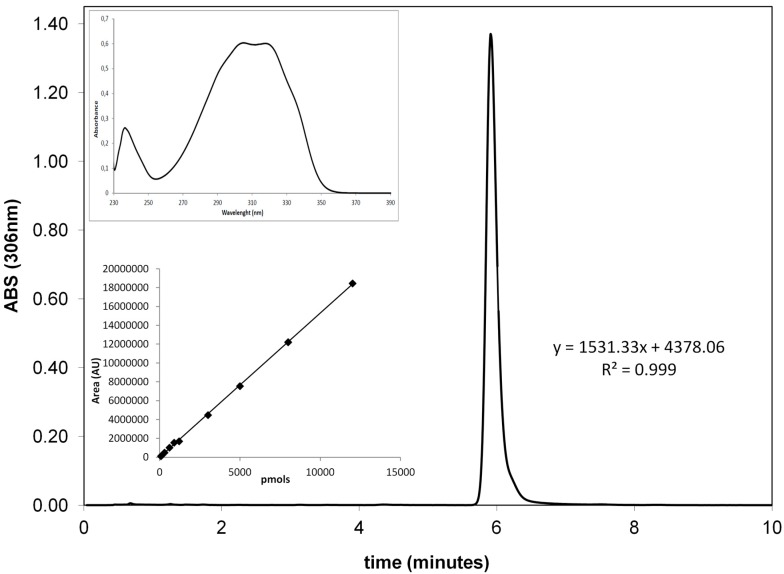
Resveratrol determination and quantitation. Resveratrol representative HPLC chromatogram. Inset: resveratrol UV-visible (UV-Vis) absorption spectrum as registered by photodiode array detector and calibration curve. The curve was obtained by plotting the resveratrol amount injected onto the HPLC and its peak area at 306 nm.

Calibration curve from 75 picomoles to 12 nanomoles was prepared by diluting in appropriate solvents a 20 mM stock solution of resveratrol in dimethylsulfoxide (DMSO). The curve (six data points, in duplicate) is linear with an *R*^2^ value of 0.999 (Figure 8). Peak detection (retention time: 6 min) and purity were checked with the photodiode array detector to verify the characteristic absorption spectrum of the polyphenol (Figure 8, inset).

### 3.5. Folin-Ciocalteau Assay

The Folin-Ciocalteau method for qualitative analyses of resveratrol in the nebulized material was set up by trapping the aerosol nebulized samples on tissue paper filters. The filters were sprayed with a solution of 30% NaCO_3_ in water and, after a 3 min incubation, the filter paper was soaked with Folin–Ciocalteau reagent. After an overnight incubation at room temperature in the absence of light, a deep blue color developed in the presence of the polyphenol.

### 3.6. GFC Analyses of CM-Glucan

Chromatographic CM-glucan analysis was performed with a TSK-G3000SW (7.5 mm × 300 mm) gel filtration column, with the same HPLC apparatus previously described, by using water as the mobile phase at a flow rate of 0.7 mL/min. For the determination of CM-glucan molecular weight, a calibration curve was obtained by analyzing 200 µL of a 1 mg/mL mixture of Pullulans analytical standards (Sigma-Aldrich, Milan, Italy) with different molecular weights. Their retention times were determined spectrophotometrically by post-column phenol-sulfuric acid derivatization by a modification of the Mazuko et al method [26]. Briefly, fractions of 0.5 mL were collected and aliquots of 50 µL were added to 150 µL of concentrated sulfuric acid (95%) in a polypropylene 1.5 mL tube. Subsequently, 30 µL of 5% phenol in water were added. After incubating at 90 °C for 5 min, the vials were cooled to room temperature in a water bath and 100 µL was transferred to a 96-well microplate. The optical density of each well was determined at 490 nm with a reference at 690 nm using a microplate reader (Appliskan microplate reader, Thermo Scientific, Vantaa, Finland).

**Figure 9:**
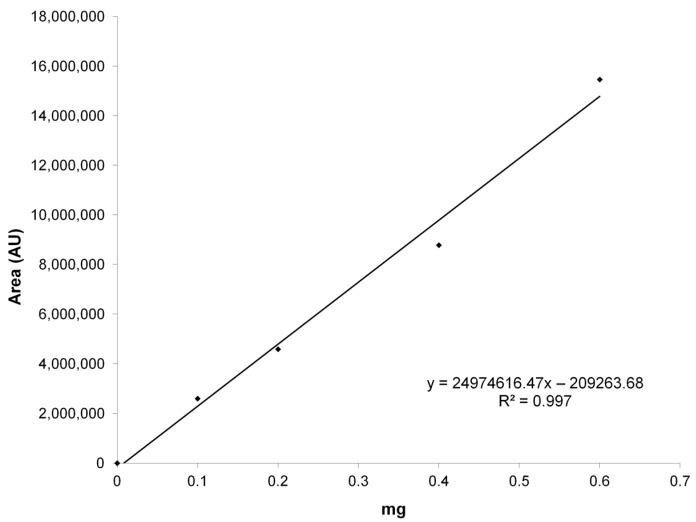
CM-glucan calibration curve. The curve was obtained by plotting the CM-glucan amount injected onto the HPLC and its peak area at 220 nm.

CM-glucan detection was performed with a photodiode array detector (PDA) by monitoring the UV absorbance of -COOH moiety at 220 nm. For CM-glucan quantitative analysis, a calibration curve (Figure 9) in the range 0.1–1 mg was prepared by replicate injections of CM-glucan at different concentrations (*R*^2^ = 0.99).

### 3.7. MTT Cell Viability Assay

Resveratrol was dissolved in DMSO at a concentration of 50 mM and then diluted in cell culture media. CM-glucan was first dissolved in water at a concentration of 10 mg/mL and then diluted to the final desired concentrations in cell culture media. HeLa cells were cultured at 37 °C in 5% CO_2_ atmosphere in Eagle’s Minimum Essential Medium (MEM, HyClone) with 10% fetal bovine serum (FBS) containing gentamicin and 4 mM glutamine. Cell viability, in the presence or absence of resveratrol or resveratrol/CM-glucan, was determined by using the 3-(4,5-dimethylthiazol-2-yl)-2,5-diphenyl tetrazolium bromide (MTT) dye reduction assay. Briefly, cells were seeded in 96-well plates at a concentration of 19,000 cells per well in order to obtain cells in sub-confluent conditions after 48 h. Cells were treated with different concentrations of resveratrol or resveratrol/CM-glucan maintaining the molar ratio used for all other experiments (1:2 *w*/*w*) and incubated at 37 °C in 5% CO_2_ atmosphere for 24 h. After treatment, 20 μL of a 5 mg/mL solution of MTT in PBS was added per well, and cells were incubated at 37 °C for 2 h. The supernatants were then removed and formazan crystals were dissolved in 100 μL of DMSO. The absorbance of each solution was determined at 570 nm with a reference at 690 nm using a microplate reader.

## 4. Conclusions

Glucans are an extremely interesting group of biopolymers endowed with many different pharmaceutical and pharmacological properties. Their use in pharmaceutical preparations and in the food industry is steadily increasing.

During the last years, our group has been studying the pharmaceutical and pharmacological properties of resveratrol and evaluated possible synergies with other bioactive molecules, in particular with carboxymethylated β-glucan. This association was found to be effective in preventing resveratrol degradation in water and in counteracting rhinovirus infection in human nasal epithelia by altering virus replication and the expression of inflammatory mediators in nasal epithelia. Its use as a nasal spray in upper respiratory tract infections was demonstrated to be safe and effective [14,15,16]. The positive interaction between β-glucan and resveratrol is also supported by a number of studies showing a significant synergy of the two compounds in stimulating the immune system [18] and in strongly reducing stress-related symptoms, including corticosterone levels and IL-6, IL-12, and interferon (IFN)-γ production compared to that of its individual components [27].

Inhaled aerosol therapies are the mainstay of treatment of lower respiratory tract diseases such as viral infections, allowing for high local drug concentrations. The aim of the present work was to develop an aerosol formulation containing an association of resveratrol and CM-glucan which could reach the lower respiratory tract. Our data demonstrate that it is possible to develop an aerosol product containing s a stabilized form of resveratrol. The particle size (expressed as MMAD) is of critical importance as the drug delivery depends on this to a major extent. We demonstrated that resveratrol/CM-glucan association results in the finest and most monodispersed particles when compared with the two single components. The association also evidenced lower values for all particle size distribution parameters suggesting that the pharmacological synergy, observed in previous studies, may be accompanied by a pharmaceutical one. Moreover, we showed that the CM-glucan matrix did not exert an inhibitory effect on resveratrol nebulization, demonstrating the good suitability of these two molecules in association for simultaneous aerosol volatilization. All these features make the resveratrol/CM-glucan association helpful for aerosol treatment of patients requiring antiviral and/or anti-inflammatory medication for lower respiratory tract diseases.

## Figures and Tables

**Table 1:** Particle size distribution of liquid formulations of resveratrol and/or CM-glucan. MMAD = mass median aerodynamic diameter; GSD = geometric standard deviation.

Sample	MMAD	GSD	Mode	D90	D10	D50	Span (D90 − D10)/D50
Resveratrol	3.28	1.488	3.60	5.37	1.89	3.24	1.07
CM-glucan	2.96	1.506	3.19	5.04	1.70	2.99	1.11
Resveratrol/CM-glucan	2.83	1.364	2.69	4.65	1.88	2.83	0.97

## Abbreviations

RP-HPLC	reversed phase high pressure liquid chromatography
GFC	gel filtration chromatography
MMAD	mass median aerodynamic diameter
GSD	geometric standard deviation
HBEC	Human Bronchial Epithelial Cells
MTT	3-(4,5-dimethylthiazol-2-yl)-2,5-diphenyltetrazolium bromide
